# The influences of the Big Five personality traits on academic achievements: Chain mediating effect based on major identity and self-efficacy

**DOI:** 10.3389/fpsyg.2023.1065554

**Published:** 2023-01-27

**Authors:** Hui Wang, Yuxia Liu, Zhanying Wang, Ting Wang

**Affiliations:** School of Business, China University of Political Science and Law, Beijing, China

**Keywords:** personality traits, professional identity, self-efficacy, academic achievement, Big Five

## Abstract

This study mainly verified the influences of personality traits on students’ academic achievements and assessed the mediating effects of major identity and self-efficacy, under the classical model of chain mediating effects, with the data of business major students. The results show that both extraversion and conscientiousness have positive total effect on students’ academic achievements, and they are mainly realized through the chain mediating effects of self-efficacy and major identity to self-efficacy, and are mainly based on self-efficacy mediating effect, which is more obvious in the dimension of behavioral efficacy. Openness also affects academic achievement through a self-efficacy mediating effect and major identity to self-efficacy chain mediating effect, but the degree of influence is weak, and the total effect is not significant; the overall effect of agreeableness personality on academic achievement is negative, and it is mainly reflected through direct effect. This finding suggests that academic achievement does not reflect students’ team ability and performance.

## 1. Introduction

College students, as successful candidates in the examination-oriented education in the college entrance examination, have already been fully tested for their basic abilities and qualities. However, the students’ learning content and form are greatly influenced by their chosen major after entering the university. If the students’ own personality and professional characteristics cannot form a good match, it may cause college students to be tired of learning and abandon their studies, reduce their academic investment, leading to a decline in academic performance and lack of competitiveness in the future talent market. College students are the country’s valuable reserve force of talents. It is of great strategic significance to improve the academic level of college students and the quality of personnel training to promote the sustained and healthy development of the national economy and the smooth upgrading and transformation of industry. Therefore, the Ministry of Education has proposed an overall goal of developing high-quality education, so that better matching results between students’ personality characteristics and major subjects studied and higher matching efficiency are the basis for developing high-quality higher education.

While the Ministry of Education is supervising and upgrading the academic achievements of college students, in fact, students also attach great importance to their own academic achievements. After entering the university stage, although the students are released from the pressure of “one exam for life”, achieving good academic achievement is still one of the most important goals for them.

The level of academic achievement is not only related to the students’ mental health, but also affects their changing role as they move from campus to workplace and their subsequent career adaptation. The study found that students with low academic achievement, worried about failing the final exam and not earning a graduation certificate, suffered tension, anxiety, and insomnia, with a concomitant, long-term influence on their mental health (Yan, 2020). In addition, those with high academic achievement can complete the transition from study to work more smoothly and gain a more favorable position in the labor market (Mike et al., 2014). However, because college students pay more attention to their own autonomy, which is manifestly different from their learning style in primary and secondary school, many students have poor learning effect and low academic achievement due to their difficulty in adapting to a more advanced college learning style (Zhao, 2019).

A growing number of studies have shown that personality traits are one of the important factors affecting college students’ academic achievements (Lei et al., 2011; Harsha et al., 2015). Personality, as a stable psychological quality, plays an important role in students’ own academic achievements. Studies have found that emotional stability can hinder students’ learning achievement, while conscientiousness can promote higher learning achievement (Chamorro-Premuzic and Furnham, 2003). It is worth noting that, although previous studies have emphasized the important role of students’ own factors in their academic achievements, in fact these studies are mainly focused on the exploration of intelligence factors, while less on non-intelligence factors such as personality traits. In addition, how personality traits affect college students’ academic achievements, and its internal mechanism of action is very worthy of our in-depth thought and exploration.

Previous studies on the influences of personality traits on academic performance often focused on the role of personality dimension traits and their transmission pathways, and generally regarded students’ self-efficacy as the main intermediary transmission pathway and formed a relatively rich and consistent body of research evidence; however, the specific self-efficacy result formed by personality traits is itself a complex process, which contains multiple influencing factors and forming paths. For example, students who score higher in certain dimensions of personality traits may obtain higher efficacy because of their personality traits, and a certain matching relationship may be formed between personality traits themselves and majors, which may further affect the results of their self-efficacy. On the basis of using self-efficacy as the main intermediary transmission path in the past, this study further explores the matching relationship between personality traits and specific majors and discusses the formation of self-efficacy from the perspective of major identity, as well as the ultimate impact on academic performance.

The remainder of the article is arranged as follows: the second part covers a review of the available literature; the third part introduces the theoretical model and measurement method used in this research; the fourth part explains the data and variables used; the fifth part presents and discusses the test results; the sixth part summarizes, and explains the limitations of the research.

## 2. Literature review

### 2.1. Effects of personality traits on academic achievement

Much of the literature shows that personality traits affect individual education level and academic achievement. Kifer (1975) was the first to explore the relationship between personality traits and academic achievement: at the same time, he also discussed the family factors in the formation of personality traits. Cunha et al. (2010) estimated the influences of cognitive ability and personality characteristics on the education level of adolescents and found that both explained the difference of 16% and 12% in the sample’s level of education respectively. Vedel (2014) summed up the 2l categories of empirical research into the “Big Five” personality characteristics and average performance (GPA) and demonstrated that the significant positive impact of conscientiousness is reflected in all the studies, while the negative impact of emotional stability is relatively common. In addition, some studies found that agreeableness and openness have certain positive impact on average performance, but the significance is weak, while extraversion has no significant impact. Noftle and Robins (2007) showed that personality traits exert different effects on the scores of psychology majors and non-psychology majors in a study using data about American undergraduates. For psychology majors, strictness has the greatest positive impact, followed by openness, emotional stability, and agreeableness. For students with all majors, the positive effects of strictness and openness are still steady, while the effects of other personality traits are no longer significant. The influences of personality characteristics on academic performance may be related to the major studied. On this basis, the scholars further investigated the influences of some more specific personality characteristics on learning achievement. Chamorro-Premuzic and Furnham (2003) found that professionalism, sense of responsibility and self-discipline under a strict dimension have a particularly significant impact on the level of education. Students with high-level ability in these aspects are more likely to emerge in the class. Duckworth and Seligman (2005) also proved that the difference in average scores that self-discipline can explain is more than twice of IQ, and after controlling IQ and previous average scores, the effect of self-discipline remains significant.

### 2.2. The influence mechanism of personality traits on academic achievement

While more evidence show that personality traits can have an important impact on students’ academic achievements, researchers have begun to pay attention to the reasons and mechanisms behind this phenomenon. There are mainly two different paths. One is represented by economists, who believe that the level of personality traits with certain characteristics or dimensions is regarded as another kind of human capital different from traditional human capital such as cognitive ability, namely new human capital, or can be called non-cognitive ability. In this way of thinking, researchers pay more attention to the different characteristics of individual thinking and ways of doing things that personality traits can imbue, as well as the resulting different academic achievements. The most important research object of this feature is self-efficacy.

Relevant research results indicate that academic self-efficacy plays an intermediary role in the relationship between conscientiousness or openness in the Big Five personality and academic achievement (Laura et al., 2013). Although there is a difference between the two: general self-efficacy is at the top, which refers to an individual’s general belief that he can successfully deal with challenges from different environments or deal with new things (Li et al., 2019); however, academic self-efficacy is at the bottom, which refers to a specific belief that an individual can successfully complete academic tasks (Wang and Miao, 2012). Caprara et al. (2011) used the data of Italian junior and senior high school students to verify the influence and mechanism of Big Five personality traits and self-efficacy on their academic achievements. Openness and self-efficacy can improve students’ academic achievements, which in turn can improve their self-efficacy and form a positive interaction. However, students need not only cope with the challenges brought about by their studies, but also to face the influence of interpersonal relationships. The study found that parent-child relationship and dormitory interpersonal relationship can significantly predict college students’ academic achievement (Pan and Gao, 2017; Chen, 2018). Therefore, individuals’ belief in their ability to meet different environmental challenges can better describe the relationship with academic achievement.

Previous studies have shown that personality can predict individual self-efficacy. Studies by Jin Dan et al. found that among the five factors of Big Five personality, extraversion, emotional stability and openness have a significant positive correlation with college students’ sense of entrepreneurial self-efficacy (Jin and Huang, 2019). When studying the relationship among academic procrastination, personality and general self-efficacy of junior high school students, Jason Zhang discovered that general self-efficacy is positively correlated with extraversion, agreeableness, openness and caution, and negatively correlated with emotional stability, (Jason, 2012) Shen Zhengfu et al. found that there is a significant positive correlation between introversion and self-efficacy in college students’ personality traits, while there is a significant negative correlation between emotional stability and self-efficacy (Shen et al., 2013).

In addition, general self-efficacy can positively predict students’ academic achievements. In fact, students’ sense of self-efficacy is more predictive of their academic achievement than academic factors closely related to academic achievement, such as learning motivation (Cao and Zhang, 2013). Previous studies implied that academic self-efficacy is a certain belief and cognition of college students towards their studies, and there is a certain correlation between academic self-efficacy and their academic achievements (Ke et al., 2015). General self-efficacy is found to positively predict students’ academic achievements (Du et al., 2010; Yang, 2016).

### 2.3. Impact of major identity on self-efficacy

In addition to considering personality traits as a universal ability, psychologists and educators are more inclined to regard personality traits as a neutral feature. There is no distinction between high and low, but depends on different cultures, organizations, positions and the degree of matching between professional and individual personality traits. Therefore, educators are eager to study which personality traits can achieve better results for a specific class of professional students. Under the guidance of this thought, researchers generally employ major identity to reflect the degree of matching between students and majors and use this as antecedent variable to study the impact on academic self-efficacy, academic input, academic achievement and other results.

On the one hand, many studies have shown that college students’ major identity is closely related to their learning input. As Chen (2013) pointed out, for normal university students, the more they identify with their major, the more willing they are to study hard. Research by Wang and Sun (2015) shows that medical college students have medium major identity and average learning input; moreover, the more they identify with their major, the harder they study. Emotional major identity has the greatest predictive effect on learning input, that is, the more college students like their major, the more time and energy they spend on their professional study. A survey conducted by Liu et al. (2014) found that college students who study clinical medicine are more invested in their studies and are more satisfied with their major, and there is a significant positive correlation between the two. Cui (2013) found that college students’ evaluation and views of their majors, professional emotions and feelings about how well their majors match their own conditions will all affect their efforts when studying and learning. The more students identify with their majors, the more they like to learn, and the more they invest in learning. Zhang and Wang (2018) also revealed a positive correlation between their major identity and learning input in the study of free normal students in special education, i.e. normal students with stronger value identity for their major, higher receptiveness to teaching and higher self-efficacy will invest more energy and time in professional learning. These studies show that major identity can positively predict the learning input of college students. This is mainly because the current college education is mainly professional education, and major identity is the basis of learning. The deeper the college students’ understanding of their major and the more positive their emotional experience, the more conscious their professional learning behavior will be, and then they will be more actively involved in learning and be happy.

On the other hand, major identity can affect the individual’s emotion, thinking and behavior. When the individual’s major identity is stronger, it will generate more willingness to demonstrate professional values and hold a positive evaluation on their own professional knowledge and skills, to improve the individual’s sense of self-efficacy. Combining the research on the relationship between major identity and self-efficacy at home and abroad, most of them are specifically aimed at medical and nursing college students. The results show that the two are significantly correlated. For example, Tao (2016) conducted a survey on the nursing major of the five-year higher vocational education and found that the students with stronger sense of self-efficacy had higher major identity. Li and Jiang (2012) investigated the self-efficacy of undergraduate nursing students and found that students with higher self-efficacy also had higher major identity, and the more they liked their major, the higher their professional skills, and they would also attempt to find positive solutions to the difficulties in professional learning. Ma and Cao’s (2016) survey of medical freshmen shows that there is a two-way positive correlation between major identity and self-efficacy, i.e. the more college students accept their major, the stronger their self-efficacy; moreover, college students with stronger sense of self-efficacy have more confidence in their major and more recognition of their major. The two affect and promote each other. Han (2014) also found that the higher the level of major identity, the stronger the self-efficacy of college students. Other scholars added the third variable to examine the relationship between major identity, self-efficacy and other variables. For example, Qie (2014) discovered that major identity not only affects self-efficacy, but also influences their achievement motivation together with self-efficacy. The aforementioned research results show that major identity and self-efficacy affect each other, which is mainly because if a college student has a strong will to his major and future career, they will fully tap his potential, highly trust his professional skills and improve their self-efficacy.

From this literature review, personality traits can be seen to have a significant impact on students’ academic performance, which has become a more common consensus in the academic community. The mechanism of this impact mainly comes from two aspects: the improvement of academic achievement brought by certain personality traits proposed under the new human capital theory; the other is the psychological and spiritual incentive effect brought by the matching of personality traits with certain majors. In these two paths, personality traits will affect students’ final academic achievement through their self-efficacy, but the relationship between them is unclear. In addition, the matching model between personality traits and professional characteristics is also a complex problem, therefore, this study mainly focuses on a specific class of professional students, avoiding the complicated problem of the matching relationship between personality traits and majors, and focusing on discussing the results of major identity formed by students’ personality traits in a specific professional background, and aiming at the double intermediary chain effect between major identity and self-efficacy, in an attempt to provide new empirical evidence related to this topic.

## 3. Models and methods

**Figure 1:**
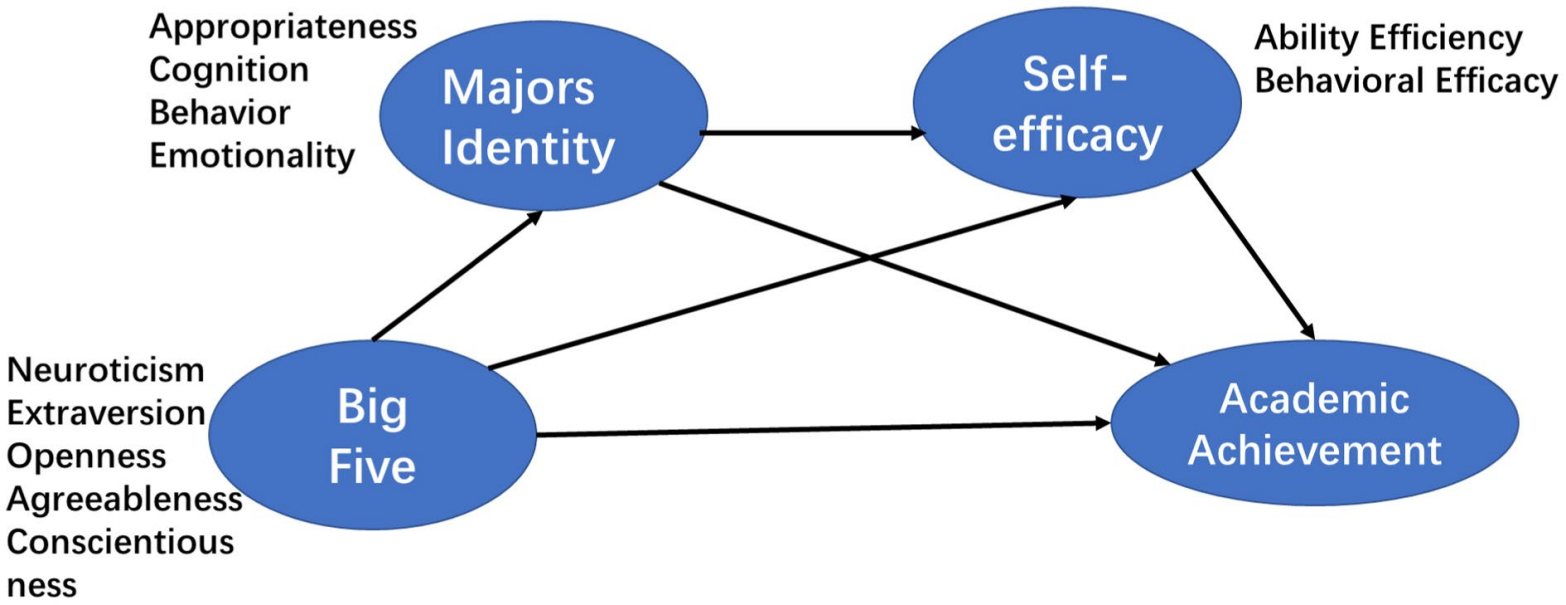
Chain mediating model of personality traits, major identity, self-efficacy and academic achievement.

The theoretical model used in this study is shown in Figure 1. According to the previous research summary, the conscientiousness in the Big Five personality traits can often have a positive direct effect on students’ academic achievement, and can have a positive indirect effect on academic achievement by improving self-efficacy, while the influence of other dimensions of personality traits is uncertain, depending on the specific situation, culture, and professional background; major identity can often produce higher self-efficacy and have a positive impact on academic achievement; the mechanism of influence of personality traits on major identity is uncertain, but the matching results between personality traits and majors can be measured for students of specified majors in specific environment. Therefore, on this basis, this study will first test the influences of personality traits on major identity, academic self-efficacy and academic achievement, then analyze the single mediating effects of major identity and self-efficacy. Finally, the joint chain mediating effects of the two are tested and compared to observe the different paths and effects of measuring the influences of personality traits on academic achievement.

In the specific measurement strategy, we first construct a structural equation model for each of the Big Five personality traits, major identity, self-efficacy, and academic achievement, and obtain the impact effects among each variable. Then we use these effect estimation results to calculate the mediation effects of major identity and self-efficacy variables and the chain mediation effects generated by both variables and measure the statistical significance of each effect through 200 repeated samples by bootstrap method. The calculation method of each effect is as follows:


(1) $$D_{pa}=\beta_{pa}$$

(2) $$I_{i}=\beta_{pi}\cdot \beta_{ia}$$

(3) $$I_{e}=\beta_{pe}\cdot \beta_{ea}$$

(4) $$I_{ie}=\beta_{pi}\cdot \beta_{ie}\cdot \beta_{ea}$$

(5) $$T_{pa}=D_{pa}+I_{i}+I_{e}+I_{ie}$$

**Figure 2:**
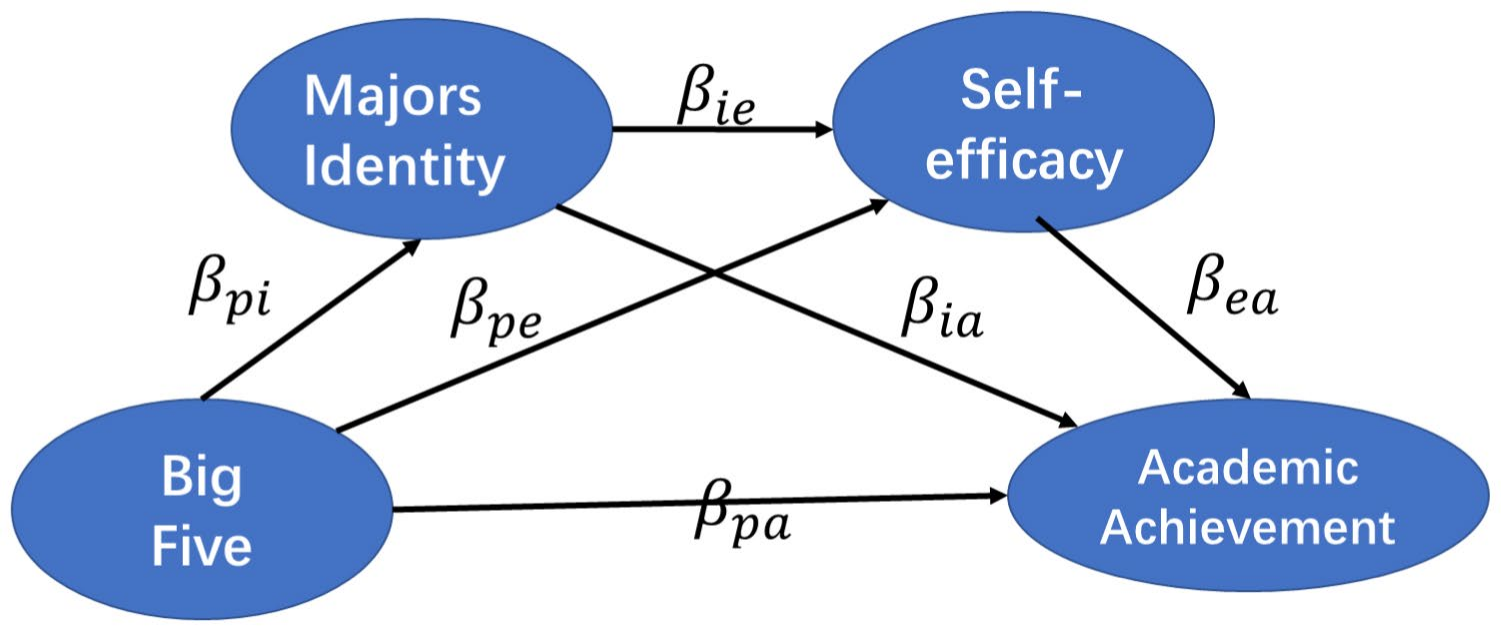
Chain-mediated effect measurement model.

$D_{pa}$ is the direct effect of personality traits on academic achievement, $I_{i}$ is the independent mediating effect of major identity, $I_{e}$ is the independent mediating effect of self-efficacy, $I_{ie}$ is the joint chain mediating effect of major identity and self-efficacy, and $T_{pa}$ is the total effect of personality traits on academic achievement. The relationships among all the effects are showed in Figure 2.

## 4. Data and variables

### 4.1. Research data

The data used in this study were collected from a questionnaire survey conducted by a business school of a university in Beijing in 2022 for sophomores and juniors. Using this data has several advantages: first, the sample is highly concentrated in terms of age structure and basic ability, which can avoid the interference of many unnecessary factors and reduce the risk of missing variables; secondly, the samples are in the same academic environment, which can make the effect model of personality traits on academic performance more evident; thirdly, it can enable us to discuss the impact matching relationship between students’ personality traits and business majors in a single business major background, simplifying our research.

### 4.2. Research variables

The key variables of the data used in this study are shown in Table 1. Among them, the students’ academic achievement is obtained by the students themselves evaluating the ranking results of their academic achievements in the major and conducting reverse value selection. The higher the value of this variable, the higher the ranking of the major that the students evaluate. The Big Five Personality Scale adopted the classic five-factor personality scale (NEO-FFI, NEO Five-Factor Inventory), with a total of 60 items (McCrae and Costa, 1991) Major identity is measured using the “College students’ major identity scale” developed by Qin (2009), with 23 items, which are divided into four dimensions: appropriateness, cognition, behavior, and emotionality, among which appropriateness refers to the degree to which students think they are suitable for the major, cognition refers to the degree to which students know their major, behavior refers to the degree to which students work hard to learn that major, and emotionality refers to the degree to which students’ take their approval of their chosen major to heart (the detailed questionnaire survey items could be seen in the Appendix). With reference to the academic self-efficacy scale compiled by Liang and Zhou (2000), academic self-efficacy is divided into two independent dimensions: ability efficiency and behavioral efficacy. Ability efficiency refers to an individual’s estimation of whether they have the ability to complete studies, achieve good results and avoid academic failure; behavioral efficacy refers to the students’ evaluation of whether their learning behavior can achieve their learning goals, and is the estimation of their behavior results. The control variables include demographic variables such as gender, as well as variables such as physical and mental health level, registered permanent residence type, parents’ education level, family economic level, only child, professional entry mode, and college entrance examination enrolment area that may affect academic achievement.

**Table 1:** List and definition of variables.

Variable category	Variable name	Number of items/Units	Definition
Interpreted variable	School achievement	Percentile	Students evaluate their percentage of professional rankings by taking the opposite value.
Key variable	Emotional stability	Index	Measured by the Big Five Personality Scale
	Extraversion	Index	Measured by the Big Five Personality Scale
	Openness	Index	Measured by the Big Five Personality Scale
	Agreeableness	Index	Measured by the Big Five Personality Scale
	Conscientiousness	Index	Measured by the Big Five Personality Scale
	Major identity	Index	Average scores across four dimensions
	Appropriateness	Index	Measured by major identity scale
	Cognition	Index	Measured by major identity scale
	Behavior	Index	Measured by major identity scale
	Emotionality	Index	Measured by major identity scale
	Academic self-efficacy	Index	Obtained by averaging the scores of two dimensions
	Ability efficiency	Index	Measured by self-efficacy scale
	Behavioral efficacy	Index	Measured by self-efficacy scale
Control variable	Gender	2	0 = male, 1 = female
	Grade	2	0 = enrolment in 2019, 1 = enrolment in 2020
	only child	2	0 = no, 1 = yes
	Professional entry mode	2	0 = non-first choice in the college entrance examination, 1 = first choice in the college entrance examination
	The Nature of Hukou	2	0 = agricultural hukou, 1 = non-agricultural hukou
	Major	4	1 = Business Administration, 2 = International Business, 3 = Financial Engineering, 4 = Economics
	Health level	3	0 = unhealthy, 1 = average, 2 = healthy
	Mental health level	3	0 = unhealthy, 1 = average, 2 = healthy
	Father’s education	3	1 = junior high school level and below, 2 = senior high school level, 3 = undergraduate college level and above
	Mother’s education	3	1 = junior high school level and below, 2 = senior high school level, 3 = undergraduate college level and above
	Household income level	3	0 = below average, 1 = average, 2 = above average
	College entrance examination student area	3	1 = western region, 2 = central region, 3 = eastern region

### 4.3. Test of reliability and validity of the scale

The reliability and validity of each measurement variable are tested, and the items with low factor load and independent residuals are removed. The reliability and validity results of the items retained are shown in Table 2. It can be seen that the Cronbach *α* coefficient of each variable is basically above 0.8, $\chi^{2}/\mathrm{df}$ basically less than 5, the TLI and CFI are basically greater than 0.9, the RMSEA is basically less than 0.09, and the SRMR is basically less than 0.05. The retained items have good homogeneity reliability and structural validity.

**Table 2:** Reliability and validity test of key variables.

Variable name	Cronbach *α*	$\chi^{2}/\mathrm{df}$	TLI	CFI	RMSEA	SRMR
Emotional Stability	0.9051	3.796	0.961	0.972	0.076	0.030
Extraversion	0.8628	4.953	0.940	0.960	0.090	0.043
Openness	0.7681	3.622	0.936	0.957	0.074	0.037
Agreeableness	0.8360	7.214	0.886	0.932	0.113	0.047
Conscientiousness	0.8814	3.006	0.973	0.982	0.064	0.028
Major identity–relevance	0.8264	4.744	0.979	0.993	0.088	0.015
Major identity-Cognition	0.8872	2.732	0.992	0.997	0.060	0.012
Major identity–Behavioral	0.9081	2.506	0.989	0.993	0.056	0.016
Major identity-Emotional	0.9505	7.758	0.972	0.991	0.118	0.015
Academic Self-efficacy-Learning Ability	0.9481	5.231	0.969	0.985	0.093	0.025
Academic Self-efficacy-Learning Behavior	0.8007	6.982	0.929	0.957	0.111	0.051

### 4.4. Variable description statistics

The results are shown in Tables 3 and 4: the mean value of the academic achievement of the explained variables is close to 50, and the overall distribution is close to normal distribution without significant deviation. The key variables affecting the Big Five personality traits, major identity and academic self-efficacy have similar distribution, and the traits are good; the distribution of the control variables is also very consistent with the overall distribution of the schools and colleges where the sample is located. The sample is of good quality and can reflect the overall situation of the students in the schools and colleges.

**Table 3:** Description of key variables.

Variable name	Entirety				
	Obs.	Mean	SD	Min.	Max.
Academic achievement	484	55.2	26.72	0	100
Emotional Stability	484	2.758	0.812	1	5
Extraversion	484	3.264	0.701	1	5
Openness	484	2.728	0.697	1	5
Agreeableness	484	2.625	0.73	1	5
Conscientiousness	484	3.565	0.621	1	5
Major identity	484	3.282	0.798	1	5
Major identity–Appropriateness	484	3.57	0.788	1	5
Major identity—Cognition	484	3.193	0.967	1	5
Major identity—Behavior	484	3.209	0.875	1	5
Major identity—Emotionality	484	3.157	0.917	1	5
Academic self-efficacy	484	3.313	0.679	1	5
Ability efficiency	484	3.269	0.763	1	5
Behavioral efficacy	484	3.356	0.67	1	5

**Table 4:** Description of control variables.

Variable name	Entirety				
	Obs.	Mean	SD	Min.	Max.
Gender	484	0.649	0.478	0	1
Grade	484	0.519	0.5	0	1
Only child	484	0.574	0.495	0	1
Professional entry mode	481	0.198	0.399	0	1
The Hukou	484	0.775	0.418	0	1
Major					
Business administration	476	0.374	0.484	0	1
International business	476	0.275	0.447	0	1
Financial engineering	476	0.179	0.383	0	1
Economics	476	0.172	0.378	0	1
Health level					
Unhealthy	484	0.105	0.307	0	1
Common	484	0.479	0.5	0	1
Health	484	0.415	0.493	0	1
Mental health level					
Unhealthy	484	0.124	0.33	0	1
Common	484	0.267	0.443	0	1
Health	484	0.61	0.488	0	1
Father’s education					
Below high school	481	0.233	0.423	0	1
High school level	481	0.2	0.4	0	1
Bachelor degree or above	481	0.568	0.496	0	1
Mother’s education					
Below high school	481	0.264	0.441	0	1
High school level	481	0.293	0.456	0	1
Bachelor degree or above	481	0.443	0.497	0	1
Household income level					
Below average	484	0.236	0.425	0	1
Average	484	0.603	0.49	0	1
Above average	484	0.161	0.368	0	1
Part of the country					
Western	484	0.207	0.405	0	1
Midland	484	0.386	0.487	0	1
East	484	0.407	0.492	0	1

## 5. Results and discussions

### 5.1. Influence of personality traits on key variables

The effects of personality traits on academic performance, major identity and self-efficacy are shown in Table 5, among which the first and second columns show the effects of personality traits on academic achievement, the third and fourth columns show the effects of personality traits on major identity, and the fifth and sixth columns show the effects of personality traits on self-efficacy. The direct effects of each dimension of personality traits on the three outcome variables are significantly different. Among them, emotional stability has no significant impact on major identity, self-efficacy and academic performance. This is not the same as the research results pertaining to new human capital. Generally, the research results tend to think that individuals with stronger emotional stability can obtain better employment results and higher income: however, if considered only from academic performance, emotional stability is not significantly related to individual ability and academic achievement. Both extraversion and conscientiousness have significant positive effects on major identity and self-efficacy, but the direct effect on academic achievement is no longer significant, although the sign is positive, which also implies the inconsistency with the mainstream research conclusions. Openness has a strong positive effect on major identity, but a weak effect on self-efficacy; agreeableness has a negative effect on major identity and academic achievement.

**Table 5:** Influences of personality traits on key variables.

	(1)	(2)	(3)	(4)	(5)	(6)
	Academic achievement	Academic achievement	Major identity	Major identity	Self-efficacy	Self-efficacy
Emotional stability	−0.524 (1.890)	−1.535 (1.943)	0.00672 (0.0483)	−0.0193 (0.0503)	−0.0460 (0.0385)	−0.0341 (0.0380)
Extraversion	2.079 (2.505)	2.799 (2.547)	0.306*** (0.0665)	0.321*** (0.0635)	0.396*** (0.0607)	0.408*** (0.0583)
Openness	0.895 (2.350)	2.112 (2.430)	0.297*** (0.0563)	0.287*** (0.0597)	0.0875** (0.0442)	0.0752 (0.0457)
Agreeableness	−4.944** (2.137)	−4.715** (2.057)	−0.0808 (0.0502)	−0.104** (0.0499)	0.0615 (0.0409)	0.0231 (0.0396)
Conscientiousness	4.431* (2.579)	2.502 (2.721)	0.255*** (0.0645)	0.233*** (0.0644)	0.342*** (0.0517)	0.306*** (0.0528)
Female		8.376*** (2.809)		−0.111 (0.0731)		−0.0876* (0.0482)
Level 2020		0.683 (2.397)		−0.0530 (0.0626)		−0.176*** (0.0440)
Non-agricultural hukou		−0.539 (3.690)		0.164 (0.104)		0.101 (0.0704)
Only child		2.356 (2.701)		−0.128* (0.0675)		0.0695 (0.0483)
The first volunteer		5.161 (3.133)		0.236*** (0.0848)		0.158*** (0.0554)
Major		Y		Y		Y
Household income level		Y		Y		Y
Father’s education		Y		Y		Y
Mother’s education level		Y		Y		Y
Body health		Y		Y		Y
Mental health		Y		Y		Y
Part of country		Y		Y		Y
Constant	44.60*** (10.42)	26.72** (12.13)	0.758*** (0.255)	0.841** (0.331)	0.526*** (0.161)	0.656*** (0.204)
Obs.	484	470	484	470	484	470
R-squared	0.035	0.109	0.307	0.368	0.511	0.576

Robust standard errors in parentheses, ***p < 0.01, **p < 0.05, *p < 0.1.

### 5.2. Analysis of single mediating effect

#### 5.2.1. Mediating effect of major identity

The results of the single mediating effect test on major identity are displayed in Table 6 among which the test result arising from use of the three-step regression coefficient method is shown. The impact of major identity on professional performance is positive, but it does not pass the significance test. Therefore, under the single mediating effect model, although extraversion, openness, and conscientiousness all have significant positive impact on the degree of major identity, the path of personality traits influencing professional performance through major identity is not significant. This result is more obvious in the Sobel test and bootstrap test (Tables 7 and 8).

**Table 6:** Single mediating effect of major identity (three-steps test).

	(1)	(2)	(3)	(4)	(5)	(6)
	Academic achievements	Major identity	Academic achievements	Academic achievements	Major identity	Academic achievements
Major identity			3.465* (1.989)			2.200 (2.087)
Emotional stability	−0.524 (1.890)	0.00672 (0.0483)	−0.547 (1.870)	−1.535 (1.943)	−0.0193 (0.0503)	−1.492 (1.936)
Extraversion	2.079 (2.505)	0.306*** (0.0665)	1.019 (2.535)	2.799 (2.547)	0.321*** (0.0635)	2.094 (2.632)
Openness	0.895 (2.350)	0.297*** (0.0563)	−0.134 (2.489)	2.112 (2.430)	0.287*** (0.0597)	1.481 (2.554)
Agreeableness	−4.944** (2.137)	−0.0808 (0.0502)	−4.664** (2.128)	−4.715** (2.057)	−0.104** (0.0499)	−4.487** (2.061)
Conscientiousness	4.431* (2.579)	0.255*** (0.0645)	3.547 (2.582)	2.502 (2.721)	0.233*** (0.0644)	1.990 (2.714)
Woman				8.376*** (2.809)	−0.111 (0.0731)	8.621*** (2.803)
Level 2020				0.683 (2.397)	−0.0530 (0.0626)	0.800 (2.397)
Non-agricultural hukou				−0.539 (3.690)	0.164 (0.104)	−0.900 (3.710)
Only child				2.356 (2.701)	−0.128* (0.0675)	2.638 (2.713)
The first volunteer examination				5.161 (3.133)	0.236*** (0.0848)	4.642 (3.205)
Major				Y	Y	Y
Household income level				Y	Y	Y
Father’s education				Y	Y	Y
Mother’s education				Y	Y	Y
Body health				Y	Y	Y
Mental health				Y	Y	Y
Part of country				Y	Y	Y
						
Constant	44.60*** (10.42)	0.758** * (0.255)	41.97*** (10.38)	26.72** (12.13)	0.841** (0.331)	24.87** (12.10)
Observations	484	484	484	470	470	470
*R*-squared	0.035	0.307	0.042	0.109	0.368	0.112

Robust standard errors in parentheses.

***p < 0.01, **p < 0.05, *p < 0.1.

**Table 7:** Single mediating effect for major identity (Sobel test).

Major identity	(1)	(2)	(3)	(4)	(5)	(6)
	Extraversion	Extraversion	Agreeableness	Agreeableness	Conscientiousness	Conscientiousness
*a* coefficient	0.306***	0.324***	−0.081	−0.100*	0.255***	0.234***
	0.060	0.061	0.054	0.054	0.063	0.638
*b* coefficient	3.466*	2.344	3.466*	2.344	3.466*	2.344
	1.802	1.865	1.802	1.865	1.802	1.865
Indirect effect	1.060*	0.760	−0.280	−0.234	0.884*	0.549
	0.589	0.621	0.238	0.226	0.508	0.461
Direct effect	1.019	2.060	−4.664**	−4.444**	3.547	2.169
	2.415	2.492	2.146	2.166	2.511	2.567
Total effect	2.079	2.820	−4.944**	−4.678**	4.431*	2.717
	2.358	2.420	2.147	2.160	2.476	2.531
Emotional Stability	Y	Y	Y	Y	Y	Y
Extraversion			Y	Y	Y	Y
Openness	Y	Y	Y	Y	Y	Y
Agreeableness	Y	Y			Y	Y
Conscientiousness	Y	Y	Y	Y		
Other control variables		Y		Y		Y

**Table 8:** Single mediating effect for major identity (Bootstrap test).

Major identity	(1)	(2)	(3)	(4)	(5)	(6)
	Extraversion	Extraversion	Agreeableness	Agreeableness	Conscientiousness	Conscientiousness
Indirect effect	1.060*	0.760	−0.280	−0.234	0.884	0.549
	0.643	0.708	0.271	0.260	0.578	0.513
Direct effect	1.019	2.060	−4.664**	−4.444**	3.547	2.169
	2.587	2.591	2.020	2.175	2.564	2.749
Emotional stability	Y	Y	Y	Y	Y	Y
Extraversion			Y	Y	Y	Y
Openness	Y	Y	Y	Y	Y	Y
Agreeableness	Y	Y			Y	Y
Conscientiousness	Y	Y	Y	Y		
Other control variables		Y		Y		Y

#### 5.2.2. Self-efficacy mediating effect

The test results of single mediating effect on self-efficacy are shown in Table 9: the effect of self-efficacy on professional performance is significantly positive, and the improvement of self-efficacy can effectively improve students’ professional performance, which is consistent with the mainstream conclusion of relevant research. It also shows that under the single mediating effect model, self-efficacy is an effective path via which personality traits to affect academic achievements. Specifically, both extraversion and conscientiousness have significant positive effects on self-efficacy, but neither the overall effect nor the direct effect on academic achievement is obvious. Therefore, it can be concluded that extraversion and conscientiousness have weak indirect effects on academic achievement; the direct and total effects of agreeableness on academic achievement are similar, while the indirect effect is 0. Therefore, agreeableness has a negative direct effect on academic achievement but no indirect effect. These results are further verified by Sobel test and bootstrap test (Tables 10 and 11).

**Table 9:** Self-efficacy single mediating effect (three-steps test).

	(1)	(2)	(3)	(4)	(5)	(6)
	Academic achievements	Self-efficacy	Academic achievements	Academic achievements	Self-efficacy	Academic achievements
Self-efficacy			9.910*** (2.702)			8.774*** (2.877)
Emotional stability	−0.524 (1.890)	−0.0460 (0.0385)	−0.0681 (1.877)	−1.535 (1.943)	−0.0341 (0.0380)	−1.236 (1.926)
Extraversion	2.079 (2.505)	0.396*** (0.0607)	−1.846 (2.735)	2.799 (2.547)	0.408*** (0.0583)	−0.777 (2.904)
Openness	0.895 (2.350)	0.0875** (0.0442)	0.0280 (2.344)	2.112 (2.430)	0.0752 (0.0457)	1.452 (2.450)
Agreeableness	−4.944** (2.137)	0.0615 (0.0409)	−5.554*** (2.117)	−4.715** (2.057)	0.0231 (0.0396)	−4.917** (2.068)
Conscientiousness	4.431* (2.579)	0.342*** (0.0517)	1.038 (2.653)	2.502 (2.721)	0.306*** (0.0528)	−0.183 (2.722)
Female				8.376*** (2.809)	−0.0876* (0.0482)	9.144*** (2.765)
Level 2020				0.683 (2.397)	−0.176*** (0.0440)	2.229 (2.357)
Non-agricultural hukou				−0.539 (3.690)	0.101 (0.0704)	−1.422 (3.627)
Only child				2.356 (2.701)	0.0695 (0.0483)	1.746 (2.662)
The first volunteer examination				5.161 (3.133)	0.158*** (0.0554)	3.777 (3.141)
Major				Y	Y	Y
Household income				Y	Y	Y
Father’s education				Y	Y	Y
Mother’s education				Y	Y	Y
Body health				Y	Y	Y
Mental health				Y	Y	Y
Part of country				Y	Y	Y
Constant	44.60*** (10.42)	0.526*** (0.161)	39.38*** (10.67)	26.72** (12.13)	0.656*** (0.204)	20.96* (12.25)
Observations	484	484	484	470	470	470
*R*-squared	0.035	0.511	0.066	0.109	0.576	0.131

Robust standard errors in parentheses, ***p < 0.01, **p < 0.05, *p < 0.1.

**Table 10:** Self-efficacy single mediating effect test (Sobel test).

	(1)	(2)	(3)	(4)	(5)	(6)
	Extraversion	Extraversion	Agreeableness	Agreeableness	Conscientiousness	Conscientiousness
*a* coefficient	0.396***	0.415***	0.061	0.031	0.342***	0.308***
	0.0427	0.043	0.039	0.039	0.045	0.045
*b* coefficient	9.910***	9.421***	9.910***	9.421***	9.910***	9.421***
	2.4884	2.593	2.488	2.593	2.488	2.596
Indirect effect	3.926***	3.906***	0.609	0.294	3.392***	2.899***
	1.0727	1.150	0.414	0.373	0.961	0.905
Direct effect	−1.847	−1.086	−5.554***	−4.972**	1.038	−0.181
	2.5228	2.618	2.120	2.133	2.582	2.622
Total effect	2.080	2.820	−4.944**	−4.678**	4.431*	2.717
	2.358	2.420	2.147	2.160	2.476	2.531
Emotional stability	Y	Y	Y	Y	Y	Y
Extraversion			Y	Y	Y	Y
Openness	Y	Y	Y	Y	Y	Y
Agreeableness	Y	Y			Y	Y
Conscientiousness	Y	Y	Y	Y		
Other control variables		Y		Y		Y

**Table 11:** Self-efficacy single mediating effect test (Bootstrap test).

	(1)	(2)	(3)	(4)	(5)	(6)
	Extraversion	Extraversion	Agreeableness	Agreeableness	Conscientiousness	Conscientiousness
Indirect effect	3.926***	3.906***	0.609	0.294	3.392***	2.899***
	1.324	1.419	0.453	0.379	0.977	0.976
Direct effect	−1.846	−1.086	−5.554***	−4.972**	1.038	−0.181
	2.711	2.903	2.048	2.152	2.693	2.785
Emotional stability	Y	Y	Y	Y	Y	Y
Extraversion			Y	Y	Y	Y
Openness	Y	Y	Y	Y	Y	Y
Agreeableness	Y	Y			Y	Y
Conscientiousness	Y	Y	Y	Y		
Other control variables		Y		Y		Y

#### 5.2.3. Influence of personality traits on self-efficacy

Further considering our previous questions, the improvement of self-efficacy will have a strong positive effect on academic achievement, so which aspect does self-efficacy mainly come from? Is it directly brought about by the personality characteristic of new human capital, or is it brought about by the matching of individual’s own characteristic with specialty? If there are both, which is better? To investigate this problem, personality traits, major identity and self-efficacy are used to construct a single mediating effect model. The generation mechanism of self-efficacy is through the magnitude of mediating effect of major identity. The results obtained by regression three-step method are demonstrated in Table 12. Major identity has a significant positive effect on the generation of self-efficacy, and the improvement of major identity will effectively improve students’ academic self-efficacy; for business majors, extraversion and stringency will not only directly improve their self-efficacy, but also indirectly through major identity. However, the proportion of indirect effect is low, about 20% of the total effect. Similar results can be seen in the Sobel test and Bootstrap test (Tables 13 and 14).

**Table 12:** Personality traits-major identity-self-efficacy mediating effect test (three steps test).

	(1)	(2)	(3)	(4)	(5)	(6)
	Self-efficacy	Major identity	Self-efficacy	Self-efficacy	Major identity	Self-efficacy
Major identity			0.254*** (0.0418)			0.244*** (0.0415)
Emotional stability	−0.0460 (0.0385)	0.00672 (0.0483)	−0.0477 (0.0351)	−0.0341 (0.0380)	−0.0193 (0.0503)	−0.0294 (0.0342)
Extraversion	0.396*** (0.0607)	0.306*** (0.0665)	0.318*** (0.0551)	0.408*** (0.0583)	0.321*** (0.0635)	0.329*** (0.0540)
Openness	0.0875** (0.0442)	0.297*** (0.0563)	0.0120 (0.0436)	0.0752 (0.0457)	0.287*** (0.0597)	0.00526 (0.0446)
Agreeableness	0.0615 (0.0409)	−0.0808 (0.0502)	0.0820** (0.0360)	0.0231 (0.0396)	−0.104** (0.0499)	0.0484 (0.0356)
Conscientiousness	0.342*** (0.0517)	0.255*** (0.0645)	0.277*** (0.0501)	0.306*** (0.0528)	0.233*** (0.0644)	0.249*** (0.0515)
Female				−0.0876* (0.0482)	−0.111 (0.0731)	−0.0604 (0.0452)
Level 2020				−0.176*** (0.0440)	−0.0530 (0.0626)	−0.163*** (0.0413)
Non-agricultural hukou				0.101 (0.0704)	0.164 (0.104)	0.0606 (0.0594)
Only child				0.0695 (0.0483)	−0.128* (0.0675)	0.101** (0.0462)
The first volunteer examination				0.158*** (0.0554)	0.236*** (0.0848)	0.100* (0.0522)
Major				Y	Y	Y
Household income				Y	Y	Y
Father’s education				Y	Y	Y
Mother’s education				Y	Y	Y
Body health				Y	Y	Y
Mental health				Y	Y	Y
Part of country				Y	Y	Y
Constant	0.526*** (0.161)	0.758*** (0.255)	0.334** (0.168)	0.656*** (0.204)	0.841** (0.331)	0.451** (0.210)
Observations	484	484	484	470	470	470
*R*-squared	0.511	0.307	0.573	0.576	0.368	0.628

**Table 13:** Personality traits-major identity-self-efficacy mediating effect test (Sobel test).

Major identity	(1)	(2)	(3)	(4)	(5)	(6)	(7)	(8)
	Extraversion	Extraversion	Openness	Openness	Agreeableness	Agreeableness	Conscientiousness	Conscientiousness
*a* factor	0.306***	0.324***	0.297***	0.282***	−0.081	−0.100*	0.255***	0.234***
	0.597	0.061	0.564	0.058	0.054	0.054	0.063	0.064
*b* coefficient	0.254***	0.246***	0.254***	0.246***	0.254***	0.246***	0.254***	0.246***
	0.031	0.031	0.031	0.031	0.031	0.031	0.031	0.031
Indirect effect	0.078***	0.080***	0.075***	0.069***	−0.021	−0.025*	0.065***	0.057***
	0.018	0.018	0.017	0.017	0.014	0.014	0.018	0.017
Direct effect	0.318***	0.335***	0.012	0.005	0.082**	0.056	0.277***	0.250***
	0.041	0.042	0.039	0.040	0.036	0.036	0.043	0.043
Total effect	0.396***	0.415***	0.088**	0.074*	0.061	0.031	0.342***	0.308***
	0.043	0.043	0.040	0.041	0.039	0.039	0.045	0.045
Emotional Stability	Y	Y	Y	Y	Y	Y	Y	Y
Extraversion			Y	Y	Y	Y	Y	Y
Openness	Y	Y			Y	Y	Y	Y
Agreeableness	Y	Y	Y	Y			Y	Y
Conscientiousness	Y	Y	Y	Y	Y	Y		
Other control variables		Y		Y		Y		Y

**Table 14:** Personality traits-major identity-self-efficacy mediating effect test (Bootstrap test).

Major identity	(1)	(2)	(3)	(4)	(5)	(6)	(7)	(8)
	Extraversion	Extraversion	Openness	Openness	Agreeableness	Agreeableness	Conscientiousness	Conscientiousness
Indirect effect	0.078***	0.080***	0.075***	0.069***	−0.021	−0.025*	0.065***	0.057***
	0.021	0.021	0.020	0.019	0.013	0.013	0.019	0.018
Direct effect	0.318***	0.335***	0.012	0.005	0.082**	0.056	0.277***	0.250***
	0.055	0.055	0.043	0.045	0.037	0.037	0.051	0.053
Emotional Stability	Y	Y	Y	Y	Y	Y	Y	Y
Extraversion			Y	Y	Y	Y	Y	Y
Openness	Y	Y			Y	Y	Y	Y
Agreeableness	Y	Y	Y	Y			Y	Y
Conscientiousness	Y	Y	Y	Y	Y	Y		
Other control variables		Y		Y		Y		Y

#### 5.2.4. Impact of major identity on academic achievement

Here, the impact of major identity on professional performance is verified. The results of the three-step test are shown in Table 15: after the self-efficacy variable is added, the direct effect of major identity on professional performance is no longer significant. The positive effect of major identity on professional performance completely comes from the path of self-efficacy improvement, while the improvement of identity itself does not bring about the improvement of performance. It is not because personality traits and professional characteristics matched that greater academic achievement can be obtained, but because professional matching improves the sense of efficacy and effort. If only matching is possible without actual effort, then even a higher degree of matching will not have a positive impact on academic performance. Sobel test and Bootstrap test results strengthen this conclusion (Table 16).

**Table 15:** Major identity-self-efficacy-academic achievement mediating effect test (three steps test).

	(1)	(2)	(3)	(4)	(5)	(6)
	Academic achievements	Self-efficacy	Academic achievements	Academic achievements	Self-efficacy	Academic achievements
Self-efficacy			7.528*** (2.548)			7.613*** (2.695)
Major identity	4.401*** (1.653)	0.498*** (0.0451)	0.648 (2.127)	3.652** (1.785)	0.455*** (0.0465)	0.189 (2.273)
Emotional stability				9.501*** (2.718)	−0.0892 (0.0560)	10.18*** (2.707)
Extraversion				1.113 (2.396)	−0.182*** (0.0496)	2.500 (2.367)
Openness				−1.387 (3.750)	0.0284 (0.0694)	−1.603 (3.644)
Agreeableness				3.132 (2.716)	0.133** (0.0574)	2.119 (2.653)
Conscientiousness				3.795 (3.139)	0.0843 (0.0647)	3.153 (3.120)
Female				1.688 (3.041)	−0.0963 (0.0588)	2.421 (2.990)
Level 2020				−2.462 (3.700)	−0.0222 (0.0721)	−2.293 (3.703)
Non-agricultural hukou				2.061 (3.525)	−0.0258 (0.0720)	2.257 (3.472)
Only child				−0.784 (3.269)	−0.0131 (0.0676)	−0.685 (3.175)
The First Volunteer				2.925 (4.033)	0.103 (0.0828)	2.137 (3.931)
Major				Y	Y	Y
Household income				Y	Y	Y
Father’s education				Y	Y	Y
Mother’s education				Y	Y	Y
Body health				Y	Y	Y
Mental health				Y	Y	Y
Part of country				Y	Y	Y
Constant	40.76*** (5.592)	1.677*** (0.151)	28.13*** (6.965)	18.61** (7.880)	1.502*** (0.196)	7.170 (8.743)
Observations	484	484	484	470	470	470
*R*-squared	0.017	0.343	0.041	0.090	0.455	0.111

Robust standard errors in parentheses, ***p < 0.01, **p < 0.05, *p < 0.1.

**Table 16:** Major identity-self-efficacy-academic achievement mediating effect test (Sobel test and Bootstrap Test).

Academic achievements	(1)	(2)	(3)	(4)
	Self-efficacy	Self-efficacy	Self-efficacy	Self-efficacy
*a* coefficient	0.498***	0.460***		
	0.031	0.032		
*b* coefficient	7.528***	8.113***		
	2.166	2.299		
Indirect effect	3.753***	3.733***	3.753***	3.733***
	1.105	1.089	1.336	1.325
Direct effect	0.648	0.068	0.648	0.068
	1.844	1.900	2.090	2.194
Total effect	4.401***	3.801**		
	1.511	1.599		
Other control variables		Y		Y

Robust standard errors in parentheses, ***p < 0.01, **p < 0.05, *p < 0.1.

### 5.3. Chain mediating effect of major identity and self-efficacy

#### 5.3.1. Emotional stability

The results of the joint chain mediating effect analysis on emotional stability, major identity, self-efficacy and academic achievement and the Bootstrap test are shown in Table 17, among which columns 1, 2, and 3 contain the results of verification without control variables and columns 4, 5, and 6 contain the results arising from the addition of control variables. Emotional stability has neither a direct nor indirect effect on academic achievement, which is consistent with the previous analytical results.

**Table 17:** Chain mediating effect of emotional stability on academic achievement.

Variables	(1)	(2)	(3)	(4)	(5)	(6)
	Major identity	Self-efficacy	Academic achievements	Major identity	Self-efficacy	Academic achievements
Emotional stability	0.00103 (0.0602)	−0.0537 (0.0428)	−2.609 (1.651)	0.0183 (0.0602)	−0.00890 (0.0420)	−3.184* (1.648)
Major identity		0.499*** (0.0451)	0.803 (2.118)		0.455*** (0.0454)	0.280 (2.216)
Self-efficacy			7.224*** (2.544)			7.548*** (2.606)
Control variable				Y	Y	Y
Constant	3.279*** (0.167)	1.825*** (0.198)	35.83*** (8.799)	17.19* (9.942)	1.785*** (0.138)	3.296*** (0.130)
Direct effect of emotion stability			−2.609 (1.619)			−3.184* (1.693)
Indirect effect of major identity			0.001 (0.114)			0.005 (0.137)
Indirect effect of self-efficacy			−0.388 (0.354)			−0.067 (0.351)
Chain indirect effect			0.004 (0.214)			0.063 (0.218)
Total effect of emotion stability			−2.992* (1.555)			−3.183* (1.648)
Observations	484	484	484	470	470	470

Standard errors in parentheses, ***p < 0.01, **p < 0.05, *p < 0.1.

#### 5.3.2. Extraversion

The results of the joint chain mediating effect analysis on extraversion, major identity, self-efficacy, and academic achievement and the bootstrap test are listed in Table 18, among which columns 1, 2, and 3 contain the results of verification without control variables and columns 4, 5, and 6 contain the results arising from the addition of control variables. The total effect of extraversion on academic achievement is 3.842, among which the direct effect of extraversion and the indirect effect of major identity are insignificant: however, the indirect effect and chain mediating effect of self-efficacy are significantly positive, and the indirect effect of self-efficacy predominates. Therefore, extraversion will have a positive effect on academic achievement, and this effect is mainly realized through the indirect effect of self-efficacy and chain mediating effect from professional identification to self-efficacy.

**Table 18:** Chain mediating effect of extraversion on academic achievement.

Variables	(1)	(2)	(3)	(4)	(5)	(6)
	Major identity	Self-efficacy	Academic achievements	Major identity	Self-efficacy	Academic achievements
Extraversion	0.555*** (0.0516)	0.477*** (0.0450)	−2.475 (2.424)	0.536*** (0.0548)	0.453*** (0.0454)	−1.434 (2.549)
Major identity		0.294*** (0.0399)	0.982 (2.175)		0.276*** (0.0385)	0.382 (2.257)
Self-efficacy			8.986*** (2.841)			8.435*** (3.030)
Control variable				Y	Y	Y
Constant	1.469*** (0.168)	0.791*** (0.154)	30.28*** (7.408)	1.374*** (0.216)	0.828*** (0.174)	8.068 (8.828)
Extraversion direct effect			−2.475 (2.542)			−1.434 (2.445)
Indirect effect of major identity			0.546 (1.258)			0.205 (1.34)
Indirect effect of self-efficacy			4.285*** (1.398)			3.824*** (1.451)
Chain indirect effect			1.468*** (0.543)			1.247** (0.56)
Total effect of extraversion			3.825** (1.851)			3.842** (1.793)
Observations	484	484	484	470	470	470

Standard errors in parentheses，***p < 0.01, **p < 0.05, *p < 0.1.

#### 5.3.3. Openness

The results of the joint chain mediating effect analysis on openness, major identity, self-efficacy, and academic achievement and the bootstrap test are listed in Table 19, among which columns 1, 2, and 3 contain the results of verification without control variables and columns 4, 5, and 6 contain the results arising from the addition of control variables. The total effect of openness on academic achievement is 0.509, but is not significant; the direct effect is negative and insignificant; the mediating effect of major identity is positive and also insignificant; the mediating effect of self-efficacy and the chain mediating effect from major identity to self-efficacy are both significantly positive. Therefore, the open personality dimension may positively affect academic achievement through two paths: self-efficacy and major identity-self-efficacy, but the result is unstable.

**Table 19:** Chain mediating effect of openness on academic achievement.

Variables	(1)	(2)	(3)	(4)	(5)	(6)
	Academic achievements	Self-efficacy	Major identity	Academic achievements	Self-efficacy	Major identity
Openness	0.451*** (0.0565)	0.166*** (0.0508)	−3.780* (2.286)	0.400*** (0.0633)	0.139*** (0.0503)	−2.212 (2.263)
Major identity		0.441*** (0.0475)	1.525 (2.242)		0.412*** (0.0463)	0.663 (2.307)
Self-efficacy			8.377*** (2.456)			8.065*** (2.612)
Control variable				Y	Y	Y
Constant	2.052*** (0.155)	1.411*** (0.156)	32.75*** (7.607)	1.715*** (0.257)	1.258*** (0.201)	10.37 (9.301)
Openness direct effect			−3.780 (2.346)			−2.212 (2.583)
Indirect effect of major identity			0.688 (1.073)			0.266 (1.031)
Indirect effect of self-efficacy			1.390** (0.599)			1.124** (0.558)
Chain indirect effect			1.668*** (0.597)			1.331** (0.608)
Total effect of openness			−0.0342 (2.043)			0.509 (2.186)
Observations	484	484	484	470	470	470

Standard errors in parentheses, ***p < 0.01, **p < 0.05, *p < 0.1.

#### 5.3.4. Agreeableness

The results of the joint chain mediating effect analysis on agreeableness, major identity, self-efficacy and academic achievement, and the Bootstrap test are listed in Table 20, among which columns 1, 2, and 3 contain the verification results without control variables, and columns 4, 5, and 6 contain the results with control variables. The total effect of agreeableness on academic achievement is -3.864, which is significantly negative; the direct effect is -5.079, significantly negative; the mediating effect of self-efficacy is 0.813, significantly positive; however, neither the mediating effect of major identity nor the chain mediating effect of major identity to self-efficacy is significant. Therefore, too high a degree of agreeableness is not beneficial to the improvement of professional performance, which is different from the impact of agreeableness on the labor market outcomes among workers. It can be considered that professional performance reflects individual ability more, while people with higher agreeableness are more suitable for team cooperation, which also reflects from one aspect that professional performance does not fully reflect the overall ability of students.

**Table 20:** Chain mediating effect of agreeableness on academic achievement.

Variables	(1)	(2)	(3)	(4)	(5)	(6)
	Academic achievements	Self-efficacy	Major identity	Academic achievements	Self-efficacy	Major identity
Agreeableness	0.163** (0.0657)	0.119*** (0.0446)	−5.763*** (1.863)	0.0996 (0.0643)	0.0951** (0.0434)	−5.079*** (1.790)
Major identity		0.482*** (0.0447)	0.849 (2.105)		0.446*** (0.0454)	0.218 (2.215)
Self-efficacy			8.706*** (2.472)			8.552*** (2.620)
Control variable				Y	Y	Y
Constant	2.854*** (0.176)	1.418*** (0.171)	38.70*** (7.839)	2.484*** (0.273)	1.267*** (0.222)	18.32* (9.552)
Agreeableness direct effect			−5.763*** (1.798)			−5.079** (2.053)
Indirect effect of major identity			0.139 (0.387)			0.022 (0.281)
Indirect effect of self-efficacy			1.035** (0.506)			0.813* (0.46)
Chain indirect effect			0.685* (0.368)			0.38 (0.318)
Total effect of agreeableness			−3.904** (1.774)			−3.864* (1.988)
Observations	484	484	484	470	470	470

Standard errors in parentheses, ***p < 0.01, **p < 0.05, *p < 0.1.

#### 5.3.5. Conscientiousness

The results of the joint chain mediating effect analysis on stringency, major identity, self-efficacy, and academic achievement, and the bootstrap test are listed in the Table 21, among which columns 1, 2, and 3 contain the verification results without control variables, and columns 4, 5, and 6 contain the results arising from the use of control variables. The total effect of stringency on academic achievement is 4.434, which is significantly positive; direct effect and major identity effect are insignificant; The mediating effect of self-efficacy is 3.311 (significantly positive); the chain mediating effect from major identity to self-efficacy is 1.281, which is significantly positive: stringency can therefore significantly improve students’ professional performance, and this overall effect is mainly achieved through the mediating effect of self-efficacy and the chain mediating effect from professional identification to self-efficacy, with self-efficacy mediating effect as the main effect, which matches our previous analysis.

**Table 21:** Chain mediating effect of conscientiousness on academic achievement.

Variables	(1)	(2)	(3)	(4)	(5)	(6)
	Academic achievements	Self-efficacy	Major identity	Academic achievements	Self-efficacy	Major identity
Conscientiousness	0.550*** (0.0575)	0.475*** (0.0430)	0.378 (2.484)	0.508*** (0.0634)	0.428*** (0.0468)	−0.267 (2.461)
Major identity		0.340*** (0.0424)	0.615 (2.117)		0.326*** (0.0421)	0.213 (2.215)
Self-efficacy			7.342*** (2.724)			7.739*** (2.830)
Control variable				Y	Y	Y
Constant	1.320*** (0.204)	0.503***(0.143)	27.51*** (8.613)	1.242*** (0.243)	0.585*** (0.194)	7.555 (9.728)
Conscientiousness direct effect			0.378 (2.347)			−0.267 (2.477)
Indirect effect of major identity			0.339 (1.241)			0.108 (1.282)
Indirect effect of self-efficacy			3.487*** (1.230)			3.311*** (1.259)
Chain indirect effect			1.374** (0.559)			1.281** (0.583)
Total effect of conscientiousness			5.578*** (2.052)			4.434** (2.21)
Observations	484	484	484	470	470	470

### 5.4. Heterogeneity test

#### 5.4.1. Gender heterogeneity

Considering that there may be significant differences in the degree and path of personality traits influencing academic achievement through major identity and self-efficacy among different gender student groups, the chain-mediated effect test is conducted for different gender sub-samples respectively, and the results are listed in Tables 22 and 23, in which Table 22 lists the chain-mediated effect test result of the influence of Big Five personality on academic achievement in male sub-samples and Table 23 lists the result of female sample. Through comparisons, we can find that for individuals of different genders, the influence and mechanism of personality traits on academic achievement are quite different.

**Table 22:** Chain mediating effect of Big Five personality on academic achievement for male.

Variables	(1)	(2)	(3)	(4)	(5)
	Emotional stability	Extraversion	Openness	Agreeableness	Conscientiousness
Big Five personality-direct effect	−7.688*** (2.606)	−13.44*** (4.592)	−7.044** (3.367)	−9.497*** (3.236)	−1.678 (4.595)
Indirect effect of major identity	0.648 (0.829)	2.831 (2.225)	1.287 (1.350)	0.703 (0.940)	1.389 (1.946)
Indirect effect of self-efficacy	0.144 (0.493)	7.737*** (2.763)	1.584 (1.127)	1.697 (1.090)	2.751 (2.509)
Chain indirect effect	0.365 (0.494)	1.458 (0.941)	0.903 (0.781)	0.722 (0.658)	0.781 (0.844)
Big Five personality-total effect	−6.531*** (2.369)	−1.412 (3.753)	−3.271 (3.014)	−6.375** (3.050)	3.244 (3.996)
Control variable	Y	Y	Y	Y	Y
Observations	166	166	166	166	166

Standard errors in parentheses, ***p < 0.01, **p < 0.05, *p < 0.1.

**Table 23:** Chain mediating effect test of Big Five personality on academic achievement for female.

Variables	(1)	(2)	(3)	(4)	(5)
	Emotional stability	Extraversion	Openness	Agreeableness	Conscientiousness
Big Five personality-direct effect	−0.432 (2.184)	3.059 (3.041)	3.231 (3.049)	−1.621 (2.225)	1.962 (2.784)
Indirect effect of major identity	0.102 (0.429)	−0.812 (1.459)	−0.981 (1.437)	−0.0245 (0.324)	−0.759 (1.604)
Indirect effect of self-efficacy	−0.467 (0.633)	3.421** (1.741)	0.448 (0.790)	0.390 (0.691)	3.746*** (1.406)
Chain indirect effect	−0.394 (0.548)	1.468** (0.731)	2.174** (0.896)	0.0956 (0.507)	1.792** (0.830)
Big Five personality-total effect	−1.190 (2.302)	7.136*** (2.159)	4.873* (2.782)	−1.159 (2.343)	6.741** (2.619)
Control variable	Y	Y	Y	Y	Y
Observations	304	304	304	304	304

Standard errors in parentheses, ***p < 0.01, **p < 0.05, *p < 0.1.

For men, emotional stability has a significant negative impact on academic performance, and the total effect stems from the direct effect of this personality trait, while the indirect effect of major identity and self-efficacy is almost zero; for women, emotional stability has neither direct effect nor indirect effect on academic achievement, and the total effect is almost zero. It seems that women’s academic achievement will not be affected by too many emotional fluctuations, while too stable a mood among male subjects is not conducive to the final result in terms of academic achievement.

Male extraversion will have a negative direct impact on academic achievement, and at the same time, it will indirectly have a positive impact on academic achievement by improving self-efficacy. The overall results of negative direct effect and positive indirect effect seem to cancel each other out, preventing extraversion from having any significant impact on male academic achievement; women’s extraversion does not show a direct impact on academic achievement, but is manifest mainly through the independent mediating effect of self-efficacy and the chain mediating effect of major identity to self-efficacy has a positive impact on the final academic achievement results, and the independent mediating effect of self-efficacy is stronger. Therefore, men who are too extroverted do not seem to have too many positive effects on academic achievement, but they can compensate for it through the synchronous improvement of self-efficacy; women’s extraversion is more conducive to academic achievement.

Male openness is akin to extraversion, which has a negative direct impact on academic achievement, but it also seems that it can be compensated for through major identity and self-efficacy improvement. This result needs more data to verify the postulate. Women’s openness has a positive impact on academic achievement mainly through the chain mediating effect from professional identification to self-efficacy, but the overall effect remains to be further tested.

Male agreeableness is akin to emotional stability, which will have a negative effect on academic achievement, and is mainly manifest as a direct effect, but with no indirect effect through major identity and self-efficacy; women’s agreeableness does not have a negative impact on academic performance, both direct and indirect effects are not obvious.

Male conscientiousness does not seem to have a beneficial effect on their academic achievements, both direct and indirect effects are unobvious; women’s conscientiousness will not have a direct impact on academic achievement, but it can exert a positive impact on academic achievement through the mediating effect of self-efficacy and the chain mediating effect of major identity to self-efficacy.

#### 5.4.2. Self-efficacy heterogeneity

Furthermore, we tested the two dimensions of self-efficacy (ability efficacy and behavior efficacy) to measure the mediating effect mechanism and intensity of the two dimensions of self-efficacy on academic achievement. The results are shown in Tables 24 and 25, among which Table 24 shows the chain mediating effect of Big Five personality traits on academic achievement in the dimension of ability validity, and Table 25 shows the chain mediating effect of the Big Five personality traits on academic achievement in the dimension of behavior validity. The mediating effect of extraversion and conscientiousness self-efficacy is still significant when the individual’s self-efficacy level is reflected solely by ability-efficacy, but the chain mediating effect of major identity to ability-efficacy is no longer significant; on the other hand, when the individual’s self-efficacy level is reflected by behavioral efficacy, the mediating effect of extraversion and conscientiousness on academic achievement’s self-efficacy and the mediating effect of major identity to self-efficacy’s chain have been strengthened. Therefore, it can be considered that the mediating effect of extraversion and conscientiousness on academic achievement’s chain from major identity to self-efficacy is more realized through the behavioral efficacy dimension.

**Table 24:** Chain mediating effect test of Big Five personality on academic achievement (ability efficiency dimension).

Variables	(1)	(2)	(3)	(4)	(5)
	Emotional stability	Extraversion	Openness	Agreeableness	Conscientiousness
Big Five personality-direct effect	−2.998* (1.621)	0.0889 (2.361)	−1.790 (2.539)	−4.930** (2.029)	1.180 (2.441)
Indirect effect of major identity	0.0254 (0.168)	0.626 (1.357)	0.637 (1.052)	0.118 (0.277)	0.529 (1.295)
Indirect effect of ability and efficiency	−0.253 (0.248)	2.300* (1.348)	0.702* (0.425)	0.663* (0.368)	1.864* (1.113)
Chain indirect effect	0.0425 (0.181)	0.826 (0.553)	0.959 (0.597)	0.284 (0.266)	0.861 (0.585)
Big Five personality-total effect	−3.183** (1.623)	3.842** (1.793)	0.509 (2.186)	−3.864* (1.988)	4.434** (2.210)
Control variable	Y	Y	Y	Y	Y
Observations	470	470	470	470	470

Standard errors in parentheses, ***p < 0.01, **p < 0.05, *p < 0.1.

**Table 25:** Chain mediating effect test of Big Five personality on academic achievement (behavioral efficacy dimension).

Variables	(1)	(2)	(3)	(4)	(5)
	Emotional stability	Extraversion	Openness	Agreeableness	Conscientiousness
Big Five personality-direct effect	−3.550** (1.613)	−1.395 (2.421)	−2.300 (2.594)	−4.924** (2.058)	−0.600 (2.467)
Indirect effect of major identity	0.00622 (0.139)	0.349 (1.255)	0.364 (0.964)	0.0555 (0.264)	0.231 (1.190)
Indirect effect of behavioral efficacy	0.299 (0.370)	3.784*** (1.290)	1.213* (0.621)	0.658 (0.470)	3.644*** (1.166)
Chain indirect effect	0.0617 (0.226)	1.103** (0.439)	1.232** (0.487)	0.346 (0.276)	1.158** (0.454)
Big Five Personality-Total Effect	−3.183** (1.623)	3.842** (1.793)	0.509 (2.186)	−3.864* (1.988)	4.434** (2.210)
Control variable	Y	Y	Y	Y	Y
Observations	470	470	470	470	470

Standard errors in parentheses, ***p < 0.01, **p < 0.05, *p < 0.1.

## 6. Conclusions and limitations

### 6.1. Key conclusions

In this study, business majors in domestic colleges and universities were taken as the research object, and the influences of the Big Five personality traits on their academic performance were evaluated. Based on the verification of the single mediating effect of major identity and self-efficacy, the chain mediating effect from major identity to self-efficacy was tested, and the following main conclusions were found:

Emotional stability has neither general effect nor direct effect on academic achievement, nor mediating effect of major identity or self-efficacy, which differs from the impact of this personality dimension on labor market results. It can be concluded that the more the work or affairs that value personal ability and achievements, the less the impact of this personality dimension;

Both extraversion and conscientiousness have a positive overall effect on students’ academic achievements, but they are mainly achieved through the mediating effect of self-efficacy and the chain mediating effect from professional identification to self-efficacy, and are mainly achieved through the mediating effect of self-efficacy, and are mainly realized through the dimension of behavioral efficacy;

Openness can also affect academic achievement through self-efficacy mediating effect and major identity to self-efficacy chain mediating effect, but the degree of influence is weak, and the total effect is insignificant;

The overall effect of agreeableness personality on academic achievement is negative, and it is mainly reflected through direct effect, which reflects that academic achievement does not seem to reflect students’ team ability and performance well;

There are significant differences in the results and mechanisms of the influences of male and female personality traits on academic achievement: male openness and extraversion have a negative direct impact on academic achievement, but they can also be compensated through major identity and self-efficacy improvement; both agreeableness and emotional stability have a negative effect on academic achievement, and the main performance is a direct effect. The direct and indirect effects of stringency are unobvious. Women’s extraversion and conscientiousness have a positive impact on academic achievement through independent mediating effect of self-efficacy and chain mediating effect of major identity to self-efficacy, while emotional stability, openness and agreeableness have no distinct direct or indirect effects on academic achievement.

### 6.2. Research limitations

This study also has many research limitations, for example, students’ academic achievements are mainly obtained through students’ self-assessment, which will inevitably be affected by students’ self-cognition bias, resulting in inaccurate results; On the other hand, the data sample size obtained in this study is small, and the robustness of the related conclusions needs to be verified in a wider range of research; Thirdly, this study only tested the results of business majors, while the matching relationship and model between personality traits and professional characteristics have not been solved, which warrants further in-depth research in the future.

## Data availability statement

The raw data supporting the conclusions of this article will be made available by the authors, without undue reservation.

## Author contributions

HW contributed to research idea, conceptualization, writing, and revising the paper. YL contributed to data analysis, methodology, and writing the paper. ZW contributed to data collection and original draft preparation. TW contributed to theoretical construction and revising the paper. All authors contributed to the article and approved the submitted version.

## Funding

This research was supported by National Natural Science Foundation of China and the project numbers are 71874205; the Key Program of National Social Science Foundation of China and the project numbers are 20AZD071; Qianduansheng Eminent Scholar Support Program in China University of Political Science and Law; the Fundamental Research Funds for the Central Universities in the Scientific Research Innovation Project in China University of Political Science and Law and the project numbers are 22ZFG79002; and the Program for Young Innovative Research Team in China University of Political Science and Law.

## Conflict of interest

The authors declare that the research was conducted in the absence of any commercial or financial relationships that could be construed as a potential conflict of interest.

## Publisher’s note

All claims expressed in this article are solely those of the authors and do not necessarily represent those of their affiliated organizations, or those of the publisher, the editors and the reviewers. Any product that may be evaluated in this article, or claim that may be made by its manufacturer, is not guaranteed or endorsed by the publisher.

## Appendix

College Students’ Major identity Scale.

Please read the following description. According to the degree of conformity between this description and your own situation, 1 is very non-conformity, 2 is comparative non-conformity, 3 is some conformity, 4 is comparative conformity and 5 is complete conformity.

1. I know what the major requires of the learners’ quality.

2. I understand the employment situation of my major.

3. I know the position of my major in this school.

4. I know the external evaluation of my major.

5. On the whole, I know my major.

6. I am willing to do the work related to my major.

7. I have accepted my major in my heart.

8. I did not want to change my major.

9. I have a relatively positive evaluation of my major.

10. I have great confidence in the future development of my major.

11. I have positive feelings toward my major.

12. I am satisfied with the general situation of my major in our school.

13. On the whole, I like my major.

14. I often read books related to my major.

15. I will finish my professional homework in time and seriously.

16. I can listen carefully in professional courses.

17. I spend a lot of time on my major.

18. I am persistent in my study of this major.

19. I actively participate in professional related practical activities.

20. I have good professional thinking.

21. My character matches this major.

22. The major I studied can reflect my specialty.

23. I feel very relaxed when studying this major.

1–4 is entitled “fitness dimension,” which measures the degree to which students think they are suitable for the major; 5–9 is a cognitive dimension, which measures students’ understanding of their major; 10–15 is the behavioral dimension, which measures the degree of students’ efforts in professional learning; 16–23 is an emotional dimension, measuring the degree of students’ inner recognition of the major.
